# Retraction: Overexpressed tumor suppressor exosomal miR-15a-5p in cancer cells inhibits PD1 expression in CD8+T cells and suppresses the hepatocellular carcinoma progression

**DOI:** 10.3389/fonc.2024.1388383

**Published:** 2024-03-05

**Authors:** Frontiers Editorial Office

The journal retracts the 19th March 2021 article cited above.

Following publication, concerns were raised regarding the integrity of the images in the published figures. Image duplication concerns were also identified in Figure 3A of the above article and Figure 2A in Zhang D, Cai G, Liu K, Zhuang Z, Jia K, Pei S, Wang X, Wang H, Xu S, Cui C, Sun M, Guo S, Song W, et al. Microglia exosomal miRNA-137 attenuates ischemic brain injury through targeting Notch1. Aging (Albany NY). 2021 Jan 10; 13:4079-4095. https://doi.org/10.18632/aging.202373

The authors failed to provide a satisfactory explanation during the investigation, which was conducted in accordance with Frontiers’ policies. As a result, the data and conclusions of the article have been deemed unreliable and the article is retracted.

This retraction was approved by the Chief Editors of Frontiers in Oncology and the Chief Executive Editor of Frontiers. The authors agree to this retraction.

